# The sonographic quantitative assessment of the deltoid muscle to detect type 2 diabetes mellitus: a potential noninvasive and sensitive screening method?

**DOI:** 10.1186/s12902-022-01107-2

**Published:** 2022-07-27

**Authors:** Kelli A. Rosen, Anay Thodge, Amy Tang, Brendan M. Franz, Chad L. Klochko, Steven B. Soliman

**Affiliations:** 1 Division of Musculoskeletal Radiology, Department of Radiology, Henry Ford Hospital/Wayne State University, 2799 West Grand Blvd, Detroit, MI 48202 USA; 2 Department of Public Health Sciences, Henry Ford Health System, 1 Ford Place, Detroit, MI 48202 USA

**Keywords:** Type 2 diabetes mellitus, Diabetes screening, Musculoskeletal ultrasound, Deltoid muscle, Shoulder, Muscle echogenicity

## Abstract

**Background:**

In our previous published study, we demonstrated that a qualitatively assessed elevation in deltoid muscle echogenicity on ultrasound was both sensitive for and a strong predictor of a type 2 diabetes (T2DM) diagnosis. This study aims to evaluate if a sonographic quantitative assessment of the deltoid muscle can be used to detect T2DM.

**Methods:**

Deltoid muscle ultrasound images from 124 patients were stored: 31 obese T2DM, 31 non-obese T2DM, 31 obese non-T2DM and 31 non-obese non-T2DM. Images were independently reviewed by 3 musculoskeletal radiologists, blinded to the patient’s category. Each measured the grayscale pixel intensity of the deltoid muscle and humeral cortex to calculate a muscle/bone ratio for each patient. Following a 3-week delay, the 3 radiologists independently repeated measurements on a randomly selected 40 subjects. Ratios, age, gender, race, body mass index, insulin usage and hemoglobin A_1c_ were analyzed. The difference among the 4 groups was compared using analysis of variance or chi-square tests. Both univariate and multivariate linear mixed models were performed. Multivariate mixed-effects regression models were used, adjusting for demographic and clinical variables. Post hoc comparisons were done with Bonferroni adjustments to identify any differences between groups. The sample size achieved 90% power. Sensitivity and specificity were calculated based on set threshold ratios. Both intra- and inter-radiologist variability or agreement were assessed.

**Results:**

A statistically significant difference in muscle/bone ratios between the groups was identified with the average ratios as follows: obese T2DM, 0.54 (*P* < 0.001); non-obese T2DM, 0.48 (*P* < 0.001); obese non-T2DM, 0.42 (*P* = 0.03); and non-obese non-T2DM, 0.35. There was excellent inter-observer agreement (intraclass correlation coefficient 0.87) and excellent intra-observer agreements (intraclass correlation coefficient 0.92, 0.95 and 0.94). Using threshold ratios, the sensitivity for detecting T2DM was 80% (95% CI 67% to 88%) with a specificity of 63% (95% CI 50% to 75%).

**Conclusions:**

The sonographic quantitative assessment of the deltoid muscle by ultrasound is sensitive and accurate for the detection of T2DM. Following further studies, this process could translate into a dedicated, simple and noninvasive screening method to detect T2DM with the prospects of identifying even a fraction of the undiagnosed persons worldwide. This could prove especially beneficial in screening of underserved and underrepresented communities.

## Background

Type 2 diabetes mellitus (T2DM) affects approximately 463 million adults worldwide, including 34.2 million or 10.5% of people in the United States (U.S.) [1–3]. The worldwide prevalence is projected to significantly increase in the coming decades, reaching 700 million by 2045 [1, 4–6]. This disease disproportionately affects the underserved, underrepresented, impoverished, and lower socioeconomic communities, as well as those in developing countries [1–3, 7, 8]. These groups account for 79% of people with T2DM [1–3, 7, 8]. Furthermore, a staggering 232 million or 50% of people with T2DM worldwide and 7.3 million or 21.4% in the U.S. are unaware and undiagnosed [1, 2, 9]. This is secondary to the current screening methods for T2DM being inconvenient, invasive, poorly sensitive, and inaccurate [10–14]. Furthermore, when T2DM is finally detected in a patient, at the time of diagnosis, approximately one-half already have one or more irreversible complications [15].

Earlier detection of this disease is critical as T2DM leads to multiple costly serious end-organ complications, including being the leading cause of both end-stage renal disease and non-traumatic lower extremity amputations [1, 3, 5, 16]. Those with T2DM are also at approximately double the risk of death when compared to those without the disease [2]. Health expenditure worldwide for treating T2DM in 2019 was at least $760 (U.S. dollars) billion and in the U.S. alone was estimated at $327 (U.S. dollars) billion in 2017 [1, 3, 17]. However, once diagnosed, treatment of T2DM with effective blood glucose management has been shown to have significant health benefits and even reduce the risk of associated ophthalmologic, renal, and neurologic diseases by 40% [1, 3, 18–21].

Given its advantages over MRI, musculoskeletal (MSK) ultrasound (US) utilization, especially at shoulder level, has significantly increased over the past few decades [22–25]. Shoulder US is often performed on patients with T2DM, given the high prevalence of T2DM in society and the increased risk of rotator cuff pathology and adhesive capsulitis in people with T2DM [26–30]. As shown in our previously published study, a qualitatively assessed increased deltoid muscle echogenicity (subjectively elevated grayscale pixel echo intensity [GPEI]) on shoulder US was a strong predictor of T2DM and may prove useful in its detection [23]. While fatty infiltration of muscle in obese individuals is known to cause muscular hyperechogenicity [31–36], our prior study revealed that both non-obese and obese patients with T2DM manifested a still greater qualitative deltoid muscle GPEI than what we observed in obese individuals who tested negative for T2DM [23].

Therefore, in this study we aimed to evaluate if our prior qualitative findings could be validated objectively using a quantitative assessment of the deltoid muscle GPEI on US to detect T2DM. We believe that following further studies, this method could serve as an opportunistic tool in screening for and detecting T2DM with the hope of identifying some of the 232 million worldwide undiagnosed people with T2DM. This could prove to be especially instrumental for screening in underserved and underrepresented communities worldwide. Earlier detection, lifestyle modifications, and treatment of this disease, through this opportunistic screening method, may prevent or reduce the known devastating complications of T2DM and help mitigate a portion of the enormous healthcare economic burden.

## Methods

This study was performed in accordance with the ethical standards of our institutional research committee and with the 1964 Declaration of Helsinki and its later amendments or comparable ethical standards. Institutional review board approval was obtained for this retrospective study, and informed consent was waived (Henry Ford Health System IRB # 13,208, August 22, 2019). Our study complied with the Health Insurance Portability and Accountability Act.

### Selection of Obese T2DM, Non-obese T2DM, and Obese non-T2DM Cohorts

Using a random number generator, the following cohorts were randomly selected from a database of patients that were included in our previously published study [23]: 31 obese patients with T2DM, 31 non-obese patients with T2DM, and 31 obese patients without T2DM. The criteria for the selection of these patients are detailed in Table 1. These patients presented between October 2005 and November 2017. A chart review confirmed the absence of any relevant history or concomitant diagnoses such as muscle contusion, strain, paralysis, myositis, rhabdomyolysis, statin-induced myopathy or any other myopathy that could alter the sonographic appearance of the deltoid muscle. Furthermore, in the non-T2DM cohort, the chart review confirmed that there were no current or past diabetes-related diagnoses, whether acute or chronic. In all cohorts and especially in the non-obese T2DM cohort, a type 1 DM diagnosis was also excluded. Furthermore, in the T2DM cohorts, a documented diagnosis of T2DM was confirmed based on the American Diabetes Association criteria for the diagnosis of T2DM. Demographic information of age, gender, race, body mass index (BMI), insulin usage, and hemoglobin A_1c_ (HbA_1c_) level were obtained from chart review for inclusion in the previous study and were again recorded.

**Table 1:** Clinical criteria for patient selection

Cohort	Inclusion Criteria (All Within 3 Months of a Shoulder US)
**Obese withT2DM (*n*** **= 31)**	-Documented diagnosis of T2DM -HbA_1c_ level > 6.5% (48 mmol/mol) -Prescribed at least 1 T2DM medication -BMI ≥ 30 kg/m^2^
**Non-obese with T2DM (*n*** **= 31)**	-Documented diagnosis of T2DM -HbA_1c_ level > 6.5% (48 mmol/mol) -Prescribed at least 1 T2DM medication -BMI < 30 kg/m^2^
**Obese without T2DM (*n*** **= 31)**	-Never diagnosed with T2DM or prediabetes/IGT -HbA_1c_ level < 5.7% (39 mmol/mol) or none available -Never prescribed T2DM medication -BMI ≥ 30 kg/m^2^
**Non-obese without T2DM (*n*** **= 31)**	-Never diagnosed with T2DM or prediabetes/IGT -HbA_1c_ level < 5.7% (39 mmol/mol) or none available -Never prescribed T2DM medication -BMI < 30 kg/m^2^

*BMI* body mass index, *HbA*_*1c*_ hemoglobin A_1c_, *IGT* impaired glucose tolerance, *T2DM* type 2 diabetes mellitus, *US* ultrasound

### Selection of non-obese non-T2DM cohort

A fourth cohort of 31 non-obese patients without T2DM with a complaint of shoulder pain and a subsequent shoulder US examination were also randomly chosen for inclusion in the study. These patients presented between March 2009 and February 2019. The criteria utilized for the selection of these ‘normal’ subjects are also listed in Table 1.

A chart review was performed to confirm the absence of any relevant history or concomitant diagnoses such as muscle contusion, strain, paralysis, myositis, rhabdomyolysis, statin-induced myopathy or any other myopathy that could alter the sonographic appearance of the deltoid muscle. Also in this cohort, the chart review confirmed that there were no current or past diabetes-related diagnoses, whether acute or chronic. Demographic information of age, gender, and race were also documented for this cohort.

### Sonographic examinations

All shoulder US examinations from these 124 patients were performed by trained dedicated MSK sonographers, all of whom possess the registered MSK sonographer (RMSKS) designation through the American Registry for Diagnostic Medical Sonography (Rockville, MD, USA). For each patient a complete shoulder US was performed utilizing a 9-MHz linear transducer (GE LOGIQ E9 unit; General Electric Company, Milwaukee, WI, USA).

**Fig. 1:**
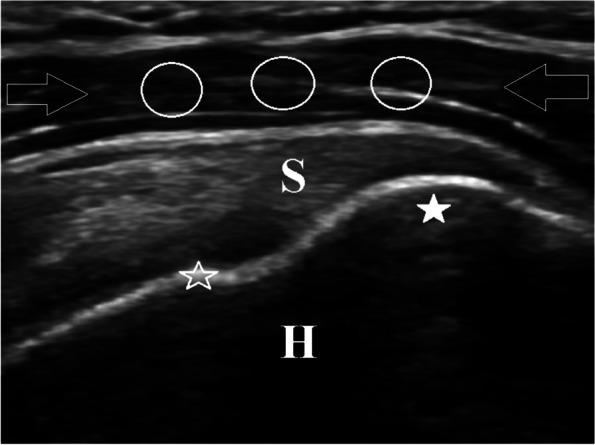
Ultrasound of a 68-year-old man without type 2 diabetes mellitus or obesity. This long-axis sonographic image of the left deltoid muscle (open arrows) is obtained at the anterior aspect of the supraspinatus tendon (S), at its insertion at the greater tuberosity (solid star) of the proximal humerus (H). The 3 circles overlying the deltoid muscle indicate the location of the grayscale pixel intensity region of interest measurements that were obtained to calculate the mean deltoid muscle value. The open star indicates the location of the single region of interest measurement obtained on the osseous cortex of the humeral head, near the anatomic neck. Notice the hypoechoic appearance of the deltoid muscle. The patient had a body mass index of 24 kg/m2. The calculated ratio (deltoid muscle/humeral cortex) for this patient was equal to 0.29, consistent with a non-type 2 diabetes mellitus status

**Fig. 2:**
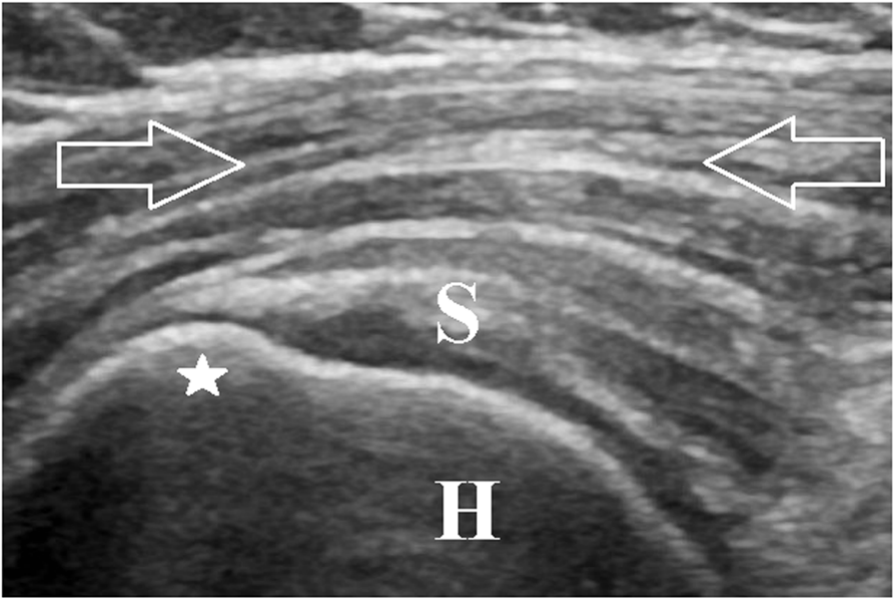
Ultrasound of a 47-year-old woman with type 2 diabetes mellitus. This long-axis sonographic image of the right deltoid muscle (open arrows) image is also obtained at the anterior aspect of the supraspinatus tendon (S), at its insertion at the greater tuberosity (solid star) of the proximal humerus (H). Notice the significant, diffusely hyperechoic (echogenic) appearance of the deltoid muscle. The patient had a body mass index of 32 kg/m2. The calculated ratio (deltoid muscle/humeral cortex) for this patient was equal to 0.67, consistent with a type 2 diabetes mellitus status

An author not involved in the blinded review of the images, for each subject, saved a single static long-axis US image of the deltoid muscle, obtained at the anterior aspect of the supraspinatus tendon, at its insertion on the greater tuberosity of the proximal humerus (Figs. 1 and 2).

All 124 images were de-identified and, using a random number generator, assigned a random number. These 124, individual, de-identified, and randomized images were then archived into a secured research survey program for the subsequent image review and measurements.

### Blinded image review and inter-observer measurements

Three MSK radiology fellows who were not involved in the selection of subjects or review of medical records independently evaluated the sonographic images quantitatively, utilizing the research survey program. The radiologists were blinded to all patients’ categories and histories. For each of the 124 patients, the three radiologists were instructed to independently measure the GPEI of a region of interest (ROI) of the deltoid muscle and a ROI of the underlying humeral cortex (Fig. 1). ROI values were automatically displayed as standard grayscale pixel levels ranging from 0 (black) to 255 (white). A hyperechoic deltoid muscle (Fig. 2) results in an increased GPEI, and therefore, a higher pixel number.

In the deltoid muscle, they were instructed to obtain 3 separate circular ROI measurements, ranging in size from 0.035 to 0.065 cm^2^, including only the deltoid muscle, without subcutaneous or subdeltoid/peribursal fat (Fig. 1). This was done to obtain an accurate representation of the entire deltoid muscle. However, to avoid areas of artifact, they were instructed to not obtain deltoid muscle measurements at the periphery of the images.

As opposed to directly measuring only the deltoid muscle GPEI, the humeral cortex, a second standard and stable anatomic location, was also measured on the same static image. This was done to ensure uniformity of the technical factors by accounting for any subtle sonographic parameter differences in image gain, depth range, or dynamic range. In regard to the humeral cortex, they were to obtain a single ROI measurement on the osseous cortex of the humeral head, along a smooth portion, near the anatomic neck. The ROI circle was to only include the bony cortex and to avoid any areas of osseous irregularity, especially along the greater tuberosity (Figs. 1 and 2).

Each of the 3 radiologists obtained these measurements on all 31 subjects from each of the 4 groups, for a total of 124 patients. These measurements were performed in a single image review session and automatically stored in the system.

### Intra-observer measurements

Following a 3-week delay to avoid recall bias, the 3 radiologists independently repeated these measurements in a single session, on a randomly selected 40 subjects from the original 124 subjects, to account for intra-observer variability.

### Sample size, power, and ratio calculations

Using measurements from each of the 3 radiologists, the ratio of deltoid muscle ROI to humeral cortex ROI for each patient was then calculated. The mean of the 3-deltoid muscle GPEI measurements was used as the numerator and the single humeral cortex GPEI measurement as the denominator (i.e., mean deltoid muscle GPEI/humeral cortex GPEI). The more hyperechoic the deltoid muscle (higher GPEI), the greater the expected ratio (Figs. 1 and 2). Sample size was calculated by the use of Power Analysis and Sample Size Software (PASS 2019) (NCSS, LLC. Kaysville, Utah, USA). The total sample size of 124 patients (31 in each group) achieved 90% power to detect difference among mean ratios using an analysis of variance F-test with a significance level of 0.05 and assuming a medium effect size of 0.35.

### Statistical analysis

Patients’ baseline characteristics were presented as mean (standard deviation) for continuous variables and frequency (percent) for categorical variables. The difference among the 4 groups was compared using analysis of variance or chi-square tests. Both univariate and multivariate linear mixed models were performed to examine the group differences in the ratio values. Multivariate mixed-effects regression models were also used, adjusting for demographic and clinical variables, and considering the variability among radiologists. Post hoc comparisons were performed with Bonferroni adjustments to identify any differences between groups. Sensitivity, specificity, accuracy, positive predictive value, negative predictive value, positive likelihood ratio, and negative likelihood ratio were also calculated based on a set ratio threshold used for the obese cohorts and a set ratio threshold used for the non-obese cohorts. Set ratio thresholds were utilized since our future studies will be aimed at translating this process into a dedicated, simple, and noninvasive screening method to detect T2DM. Therefore, set ratio thresholds were determined using Youden’s J statistic (index) with a 1:2 weight for specificity and sensitivity, respectively. This hypothetical method would require little to no training and optimally not even require an actual displayed or visualized image. By simply placing an US transducer on a person’s shoulder, potentially a dedicated low-cost portable handheld automated US unit, these automatically calculated ratios would then be automatically compared to the set ratio thresholds, depending on the person’s BMI. Subsequently, an automated probability or result would be displayed.

Both inter- and intra-radiologist variability or agreement were assessed using two‐way mixed-effects models to calculate the intraclass correlation coefficient (ICC) value for each measurement. All ICC values were interpreted using the Rosner interpretation (0‐0.40: poor agreement; > 0.40‐0.75: good agreement; and > 0.75‐1.00: excellent agreement).

All statistical analyses were performed using SAS 9.4 (SAS Institute Inc., Cary, NC, USA). Statistical significance was defined as a *P* < 0.05.

## Results

### Study cohorts

Of the 31 obese patients with T2DM, the age range was 34–78 years with a mean age of 60.7. The mean BMI was 38.7 kg/m^2^ with a range from 31–55 kg/m^2^.The average HbA_1c_ level was 7.7% (61 mmol/mol) with a range from 6.9%-11.9% (52–107 mmol/mol). Additional demographic data is listed in Table 2.――

**Table 2:** Patient demographics, BMI, HbA_1c_, insulin usage, and muscle/bone ratios among the study cohorts

Patient Data		Obese with T2DM (*n* = 31)	Non-obese with T2DM (*n* = 31)	Obese without T2DM (*n* = 31)	Non-obese without T2DM (*n* = 31)	*P*-value
Age, years (mean ± SD)		60.7 ± 17.6	65.6 ± 18.0	36.4 ± 17.7	39.6 ± 17.4	< 0.001
Gender	Female	18 (58.1%)	21 (67.7%)	14 (45.2%)	12 (38.7%)	0.01
	Male	13 (41.9%)	10 (32.3%)	17 (54.8%)	19 (61.3%)	
Race	Black	15 (48.4%)	20 (64.5%)	15 (48.4%)	10 (32.3%)	0.09
	White	16 (51.6%)	11 (35.5%)	16 (51.6%)	21 (67.7%)	
BMI, kg/m^2^ (mean ± SD)		38.7 ± 2.7	25.6 ± 7.6	33.9 ± 8.8	24.4 ± 3.0	< 0.001
HbA_1c_ (mean ± SD)		7.7% ± 1.6 (61 mmol/mol)	7.2% ± 1.5 (55 mmol/mol)	――――	――――	0.23
Insulin usage		17 (54.8%)	11 (35.5%)	――――	――――	0.13
Muscle/Bone ratio						< 0.001
	Average	0.54	0.48	0.42	0.35	
	Median	0.54	0.48	0.41	0.34	

*BMI* body mass index, *HbA*_*1c*_ hemoglobin A_1c_, *T2DM* type 2 diabetes mellitus

Categorical data is represented as frequency (percent of column). Numerical data is represented as mean ± standard deviation (SD)

Of the 31 non-obese patients with T2DM, the age range was 49–87 years with a mean age of 65.6. The mean BMI was 25.6 kg/m^2^ with a range from 19–29 kg/m^2^. The average HbA_1c_ level was 7.2% (55 mmol/mol) with a range from 6.8%-13.6% (51–125 mmol/mol). Additional demographic data is listed in Table 2.

Of the 31 obese patients without T2DM, the age range was 18–69 years with a mean age of 36.4. The mean BMI was 33.9 kg/m^2^ with a range from 30–49 kg/m^2^. Additional demographic data is listed in Table 2.

Of the 31 non-obese patients without T2DM, the age range was 18–76 years with a mean age of 39.6. The mean BMI was 24.4 kg/m^2^ with a range from 18–29 kg/m^2^. Additional demographic data is listed in Table 2.

### Ratio results and statistical significance

Overall, the deltoid muscle/bone ratio averages and medians for each group were as follows, respectively: obese T2DM, 0.54 and 0.54; non-obese T2DM, 0.48 and 0.48; obese non-T2DM, 0.42 and 0.41; and non-obese non-T2DM, 0.35 and 0.34 (Table 2).

These ratio differences demonstrated statistical significance. When compared to the ‘normal’ non-obese group without T2DM, the obese T2DM ratio was increased by 0.19 (*P* < 0.001), the non-obese T2DM was increased by 0.13 (*P* < 0.001), and the obese non-T2DM was increased by 0.07 (*P* = 0.03).

Following multivariate analysis with adjustments for age, gender, and race, the ratio differences remained statistically significant. When compared to the ‘normal’ non-obese group without T2DM, the obese T2DM ratio was increased by 0.15 (*P* < 0.001), the non-obese T2DM was increased by 0.08 (*P* = 0.04), and the obese non-T2DM was increased by 0.07 (*P* = 0.02).

### Intra- and inter-observer agreement

There was excellent inter-observer agreement between all 3 MSK radiology fellows (ICC 0.87 (95% CI 0.81 to 0.92)). Following the 3-week delayed measurements, there was also excellent intra-observer agreements (ICC 0.92 (95% CI 0.88 to 0.94), 0.95 (95% CI 0.92 to 0.97), and 0.94 (95% CI 0.90 to 0.96)).

### Sensitivity, specificity, positive predictive value, and negative predictive value

Knowing a patient’s BMI and using a threshold ratio of greater than approximately 0.43 if obese, and a threshold ratio of greater than approximately 0.36 if non-obese, the sensitivity for detecting T2DM is 80% (95% CI 67% to 88%) with a specificity of 63% (95% CI 50% to 75%). The accuracy is equal to 71% (95% CI 62% to 79%). The positive predictive value is 68% (95% CI 60% to 75%) and the negative predictive value is 75% (95% CI 64% to 83%). The positive likelihood ratio is 2.13 (95% CI 1.5 to 3) and the negative likelihood ratio is 0.33 (95% CI 0.2 to 0.6).

Moreover, using a threshold ratio of greater than approximately 0.31 if obese, and a threshold ratio of greater than approximately 0.33 if non-obese, the sensitivity for detecting T2DM increases to 94% (95% CI 84% to 98%) with a specificity of 31% (95% CI 20% to 44%). The positive predictive value is 57% (95% CI 53% to 62%) and the negative predictive value is 83% (95% CI 63% to 93%). The positive likelihood ratio is 1.35 (95% CI 1.13 to 1.61) and the negative likelihood ratio is 0.21 (95% CI 0.08 to 0.58).

### Effects of demographics, insulin usage, race, and BMI on ratios

Women, on average, had a 0.1 increase to the ratio when compared to men, which was statistically significant (*P* = 0.0036). Insulin users, on average, had a 0.02 increase to the ratio when compared to non-insulin users, albeit statistically insignificant (*P* = 0.51). Whites had a 0.07 increase to the ratio when compared to blacks, which was statistically significant (*P* = 0.035).

Furthermore, 1 unit increase of BMI (kg/m^2^) was associated with only a 0.006 increase to the ratio (*P* = 0.007). For example, the difference in mean BMI between the obese T2DM group and the non-obese group without T2DM is 14.3 kg/m^2^. This would equate to a ratio increased by 0.086 in the obese T2DM group, however, when compared to the non-obese group without T2DM, the obese T2DM group’s ratio was actually increased by an astonishing nearly 0.2 (*P* < 0.001), demonstrating that there is an additional element elevating these muscle/bone ratios, out of proportion to the just the influence of BMI. No significant ratio differences were identified when using multivariate analysis adjusting for age, gender, race, BMI and HbA_1c_ levels.

## Discussion

In our previous study, we demonstrated that a qualitatively assessed elevation in deltoid muscle echogenicity on US was both sensitive for and a strong predictor of a T2DM diagnosis [23]. In this first study of its kind, we confirmed that a sonographic quantitative assessment of the deltoid muscle GPEI, using muscle/bone ratios, is also sensitive and accurate for the detection of T2DM in both obese and non-obese cohorts.

Worldwide, an astonishing 232 million or 50% of people with T2DM are undiagnosed, including 7.3 million in the U.S. alone [1, 2, 9]. Underdiagnosis is especially prevalent in underserved, underrepresented, impoverished, and lower socioeconomic communities, as well as developing countries, which account for 79% of those affected by T2DM [1–3, 7, 8]. Earlier detection of T2DM is extremely important. If left uncontrolled, T2DM leads to multiple medically devastating and costly end-organ complications, and nearly doubles the risk of death [1–3, 5, 15, 16]. Moreover, medical expenses for treating T2DM in 2019 worldwide were at least $760 (U.S. dollars) billion, including approximately $327 (U.S. dollars) billion in the U.S. alone [1, 3, 17].

Current screening methods for T2DM are limited to blood tests, which may not be ideal since they are invasive and require the use of laboratory analysis that can be time, labor, and resource intensive. Additionally, phlebotomy, for some individuals, can be a traumatic experience that may lead to unnecessary anxiety and side effects such as ecchymosis, bleeding, vasovagal reactions, skin irritation, and pain. Furthermore, current screening methods for T2DM are lacking given their poor sensitivities, inaccuracies, inconveniences, and invasiveness [10–14].

As utilization of MSK US increases [22–25], a unique opportunity arises for detecting T2DM in undiagnosed, unsuspecting individuals presenting for [seemingly] unrelated medical care. As published in our prior study, as a large institution performing a substantial volume of MSK US and, in particular, shoulder US, it has been our experience that the incidental detection of a hyperechoic deltoid muscle has on multiple occasions resulted in the incidental diagnosis of previously undetected T2DM [23]. Following further studies, we believe these results can be translated into a new dedicated, simple, and noninvasive diagnostic screening tool for the detection of T2DM. This screening tool could result in prevention or reduction of the devastating T2DM complications and help reduce the enormous disease-associated economic burden.

### Hypotheses

Although the exact cause of the sonographically increased muscle/bone ratio in T2DM is uncertain, our findings, in combination with those of our previous study [23], offer a few hypotheses. Firstly, given that this ratio is increased in both obese and non-obese persons with T2DM, this could relate to excessive adipose muscle infiltration, out of proportion to the BMI level [34–36]. Stouge and colleagues, in a study utilizing magnetic resonance imaging, demonstrated increased fat accumulation in the muscles of patients with T2DM [37]. Furthermore, multiple studies have shown that excess adipose muscle infiltration (‘myosteatosis’ and ‘muscle lipotoxicity’) is associated with muscle insulin resistance and can affect muscle function [38–50]. However, studies performed on patients with neuromuscular diseases, including muscular dystrophies, have shown that muscle echogenicity on US can actually decrease with excessive adipose muscle infiltration, likely secondary to decreased acoustic impedance [51, 52], which would actually decrease the muscle/bone ratio.

Taking this into consideration, an alternate hypothesis is that this increased ratio could also relate to decreased intramuscular glycogen, in addition to excessive adipose muscle infiltration. It is well known that insulin resistance in T2DM results in impaired insulin-stimulated intramuscular glycogen synthesis [53, 54]. He and Kelley, in their study utilizing muscle biopsies of non-obese and obese patients with and without T2DM, demonstrated that intramuscular glycogen levels are decreased up to 65% in those with T2DM [55]. Multiple studies have also shown that decreased intramuscular glycogen levels in the postexercise state and in critically ill patients can be visualized on US as increased muscle echogenicity [23, 56–59] and therefore cause an increase in the muscle/bone ratio. Further studies are necessary to verify these potential hypotheses and identify specific causation.

#### Prediabetes/Impaired glucose tolerance and limitations

The limitations of this study should be acknowledged when interpreting the results. Firstly, given the retrospective nature of the study, we had an unequal gender and age representation in each cohort (Table 2). However, multivariate analysis failed to show any significant ratio differences when adjusting for age. Nevertheless, a future prospective investigation, controlling for gender and age, could be performed. Next, given that intramuscular glycogen depletion in the postexercise state and with dehydration have also been shown to cause a hyperechoic deltoid muscle [56–58] and, therefore, increase the muscle/bone ratio, lack of awareness of our patients’ exercise regimen and hydration status is an additional limitation. Another limitation of this study is non-inclusion of patients with type 1 diabetes and prediabetes (impaired glucose tolerances [IGT]) patients. A future prospective study could be performed with the inclusion of both persons with type 1 diabetes and prediabetes/IGT.

Interestingly however, upon a 2-year follow-up review of the non-T2DM cohort by whom the set threshold ratios for measuring sensitivity and specificity were labelled as false positives, we found 10 patients had subsequently developed prediabetes/IGT based on abnormal HbA_1c_ levels. Using this updated data, our sensitivity rose to 82%, specificity to 75%, and accuracy to 79% (versus original sensitivity of 80%, specificity of 63%, and accuracy 71%). This not only suggests that the quantitative method detects prediabetes/IGT, but also proposes that increased deltoid muscle echogenicity may predate or predict elevation of HbA_1c_ levels. This is vitally important as prediabetes/IGT affects a staggering 374 million or 1 in 11 adults worldwide, including 88 million or 34.5% of adults in the U.S. [1–3]. Furthermore, an overwhelming 90% of these patients -more than 79 million in the U.S. alone- are undiagnosed and completely unaware of their prediabetic/IGT status [1, 3], placing them at a very high risk of developing T2DM [1–3]. It is known that skeletal muscle insulin resistance is the primary defect in the development of T2DM, often occurring decades before β-cell failure and apparent metabolic dysfunction [60]. Could these identified US changes represent the noninvasive detection of early muscle insulin resistance and dysfunction, prior to clinically apparent metabolic dysfunction? Is US offering us an inexpensive, noninvasive window into the development and natural history of prediabetes/IGT and developing T2DM? And, is US identifying early muscle insulin resistance [60], prior to elevation of HbA1c levels? If further studies can confirm these hypotheses and validate the use of this screening method for prediabetes/IGT, this would prove extremely beneficial, as earlier lifestyle modifications have been shown to reduce the risk of developing T2DM by greater than 50% [1, 3, 18–21, 61, 62].

## Conclusion

In conclusion, our study demonstrates that a sonographic quantitative assessment of the deltoid muscle is both sensitive and accurate for the detection of T2DM. This method could be used during shoulder US as a supplemental opportunistic tool aiding in earlier detection of T2DM in patients who may otherwise go undiagnosed. Following further studies, this process could translate into a dedicated, simple, and noninvasive screening method to detect T2DM with the prospects of identifying even a fraction of the 232 million undiagnosed persons with T2DM and potentially the hundreds of millions of undiagnosed persons with prediabetes/IGT worldwide. This could prove especially beneficial in screening of underserved and underrepresented communities, as well as developing countries. Earlier diagnosis and therefore earlier treatment, may prevent or reduce the devastating complications of T2DM and help mitigate a portion of the enormous disease-associated healthcare economic burden.

## Data Availability

The anonymized datasets used and/or analyzed during the current study are available from the corresponding author on reasonable request.

## Abbreviations

BMI: Body mass index
GPEI: Grayscale pixel echo intensity
HbA_1c_: Hemoglobin A_1c_
ICC: Intraclass correlation coefficient
IGT: Impaired glucose tolerance
MSK: Musculoskeletal
T2DM: Type 2 diabetes
ROI: Region of interest
US: Ultrasound
U.S.: United States
